# Artificial neural network cascade identifies multi-P450 inhibitors in natural compounds

**DOI:** 10.7717/peerj.1524

**Published:** 2015-12-21

**Authors:** Zhangming Li, Yan Li, Lu Sun, Yun Tang, Lanru Liu, Wenliang Zhu

**Affiliations:** 1 Department of Pharmacy Administration, Harbin Medical University, Harbin, China; 2 Department of Pharmacy, The Fourth Hospital of Harbin Medical University, Harbin, China; 3 Shanghai Key Laboratory of New Drug Design, School of Pharmacy, East China University of Science and Technology, Shanghai, China; 4 Institute of Clinical Pharmacology, The Second Affiliated Hospital of Harbin Medical University, Harbin, China

**Keywords:** Neural network cascade, P450, Multi-P450 inhibitor, Natural compound

## Abstract

Substantial evidence has shown that most exogenous substances are metabolized by multiple cytochrome P450 (P450) enzymes instead of by merely one P450 isoform. Thus, multi-P450 inhibition leads to greater drug-drug interaction risk than specific P450 inhibition. Herein, we innovatively established an artificial neural network cascade (NNC) model composed of 23 cascaded networks in a ladder-like framework to identify potential multi-P450 inhibitors among natural compounds by integrating 12 molecular descriptors into a P450 inhibition score (PIS). Experimental data reporting *in vitro* inhibition of five P450 isoforms (CYP1A2, CYP2C9, CYP2C19, CYP2D6, and CYP3A4) were obtained for 8,148 compounds from the Cytochrome P450 Inhibitors Database (CPID). The results indicate significant positive correlation between the PIS values and the number of inhibited P450 isoforms (Spearman’s *ρ* = 0.684, *p* < 0.0001). Thus, a higher PIS indicates a greater possibility for a chemical to inhibit the enzyme activity of at least three P450 isoforms. Ten-fold cross-validation of the NNC model suggested an accuracy of 78.7% for identifying whether a compound is a multi-P450 inhibitor or not. Using our NNC model, 22.2% of the approximately 160,000 natural compounds in TCM Database@Taiwan were identified as potential multi-P450 inhibitors. Furthermore, chemical similarity calculations suggested that the prevailing parent structures of natural multi-P450 inhibitors were alkaloids. Our findings show that dissection of chemical structure contributes to confident identification of natural multi-P450 inhibitors and provides a feasible method for virtually evaluating multi-P450 inhibition risk for a known structure.

## Introduction

The human cytochrome P450 (P450) superfamily is composed of 57 heme-containing enzyme isoforms that are implicated in oxidative metabolism of a large number of endogenous and exogenous substances. P450s are responsible for approximately three-quarters of the metabolism of clinical drugs in the human body (Guengerich, 2008). However, only five P450 isoforms (CYP1A2, CYP2C9, CYP2C19, CYP2D6, and CYP3A4) are responsible for over 90% of P450-mediated metabolic elimination of clinical drugs (Williams et al., 2004).

Although most clinical drugs need P450s for oxidative metabolism and ultimately excretion from the body (Williams et al., 2004; Guengerich, 2008), the metabolic activities of P450s are often affected by large amounts of drugs or compounds (Rendic & Di Carlo, 1997; Lin & Lu, 1998; Pelkonen et al., 2008). Therefore, the risk of exposure to potential adverse drug-drug interactions (DDIs) should be seriously considered when adopting combination drug therapy (Tanaka, 1998; Lazarou, Pomeranz & Corey, 1998; Ajayi, Sun & Perry, 2000). Compared to P450 induction, inhibiting P450 enzyme activity May restrict or stop existing metabolic and elimination pathways and result in excessive exposure to co-administered drugs that undergo P450-mediated metabolism. Isoherranen et al., (2012) demonstrated that co-administration with a multi-P450 inhibitor consistently led to an extremely high blood concentration of the affected drug. A clear example of this effect is illustrated by the 128-fold increase in ramelteon exposure when co-administered with fluvoxamine, a multi-P450 inhibitor (Obach & Ryder, 2010). The above findings strongly suggest the need for more stringent assessment and clinical management of potential P450 inhibitors that simultaneously inhibit multiple drug metabolizing P450s rather than only one of them.

In addition to methodological improvements for evaluation of *in vitro* P450 inhibition by drugs and chemicals (Spaggiari et al., 2014), efforts in the past decade have also substantially advanced identification of P450 inhibitors using in silico approaches (Mishra, 2011). Recently, Cheng et al. (2011) proposed a series of virtual P450 inhibitor classifiers, each of which was designed to independently predict potential inhibition of chemicals against one of the five P450 isoforms most frequently involved in drug metabolism. This strategy applied integration of multiple computational models using different algorithms to distinguish P450 inhibitors from non-inhibitors.

Considering the higher DDI risk caused by co-administered multi-P450 inhibitor drug(s), we innovatively developed an in silico model to identify chemicals that can block multiple P450-mediated metabolic channels. Unlike the multiple solo-isoform design strategy adopted previously (Cheng et al., 2011), a simple prediction concept was implanted into our virtual multi-P450 inhibitor discriminator that aimed to efficiently assess the possibility of multi-P450 inhibition by chemicals with defined molecular structure. To accomplish this goal, we applied a novel model construction method, which we termed a neural network cascade (NNC). A NNC is a cascade of many small artificial neural networks (ANNs) structured in a ladder-like framework. Just as illustrated previously (Zhu & Kan, 2014), each small ANN in the NNC was assigned to independently fulfill a relatively simple task such as data transformation, information integration, or prediction output. As a whole, the NNC provides prediction superior to a regular ANN model.

In this study, we built a NNC with a cascade architecture of 23 ANNs to construct a virtual prediction model of multi-P450 inhibitors by translating 11 two-dimensional molecular descriptors and one three-dimensional molecular descriptors into a single parameter that perceives whether a chemical extensively inhibits drug-metabolizing P450s. This innovative virtual screening method provides a feasible approach for rapid identification of drugs or chemicals with high DDI risk.

Currently, co-use of modern and traditional medicine therapies have been accepted worldwide. It was known that the enzymatic activity of P450s could also be inhibited by natural compounds (Zhou et al., 2003). However, compared with synthetic compounds (Cheng et al., 2011), there is no knowledge about the existence and proportion of multi-P450 inhibitors in the entirety of natural compounds and their structural features. By establishing the NNC model, we had an opportunity to reveal natural compounds with high DDI risk due to multi-P450 inhibition among the approximately 160,000 monomeric natural compounds recorded in TCM Database@Taiwan (Chen, 2011). It was thought that such an effort might bring new knowledge about potential multi-P450 inhibition caused by natural compounds and contribute to rational use of natural compounds and herbs.

## Materials and Methods

### Acquisition of *in vitro* data and chemical re-sorting

The dataset of experimentally validated P450 inhibitors and non-inhibitors was downloaded from the LMMD Cytochrome P450 Inhibitors Database (CPID) (Cheng et al., 2011). Only small compounds (molecular weight < 800 Dalton) were subjected to further analysis. The P450 inhibitor and non-inhibitor classification for chemicals in the CPID followed the threshold criterion of Auld’s reports and the PubChem BioAssay database (Veith et al., 2009; Wang et al., 2009). Briefly, for chemicals in PubChem Data Set I in the CPID, a P450 inhibitor was defined for AC_50_ ≤ 10 µM whereas a P450 non-inhibitor was classified as AC_50_ > 57μM. The AC_50_ is the concentration that inhibits 50% of the activity of a specific P450 isoform. For compounds in PubChem Data Set II, P450 inhibitor was defined if PubChem activity score > 40 whereas the compound was considered a non-inhibitor for PubChem activity score = 0. A PubChem activity score > 40 indicates an IC_50_ (the concentration leading to 50% inhibition of substrate metabolism) <40μM (Wang et al., 2009). The two threshold criteria were consistent in distinguishing between inhibitors and non-inhibitors (Cheng et al., 2011). The original data were stored in ten Excel files that were merged into a single dataset, after which all the compounds underwent a unified re-sorting operation according to the number of inhibited P450 isoforms (CYP1A2, CYP2C9, CYP2C19, CYP2D6, and CYP3A4). This sorting identified 8,148 compounds with complete *in vitro* inhibition data for all five P450 isoforms(Table S1). The data for these compounds were included in this study to establish an NNC-based multi-P450 inhibitor prediction pipeline. Chemicals were categorized by number of inhibited P450 isoforms: 0, P450 non-inhibitor; 1–2, non-extensive P450 inhibitor; and 3–5, multi-P450 inhibitor (Table S2). An additional 1,919 P450 inhibitors with incomplete *in vitro* inhibition data in the CPID database but known to inhibit at least one of the five P450 isoforms were included as model application set I for model validation (Table S3).

### Mechanism-based inhibitors (MBIs) and natural compounds

A comprehensive literature search was performed in PubMed using the search terms “mechanism-based inhibition and P450” and “mechanism-based inactivation and P450”. Experimental evidence of MBIs against the P450 isoforms studied herein was extracted independently by two researchers (ZL and YL). Any disagreement was resolved by consensus. The database PubChem Compound was then used to search for the simplified molecular input line entry specification (SMILES) strings of the MBIs. If no corresponding ID was available for a MBI in PubChem Compound, the online SMILES translator (http://cactus.nci.nih.gov/translate/) was applied to generate a SMILES string based on the reported chemical structure. Additionally, the structural information for natural compounds from ZINC (Irwin et al., 2012) was downloaded from TCM Database@Taiwan (Chen, 2011), the world largest database of small molecular natural compounds. Finally, approximately 160,000 non-duplicate natural compounds were included in our study.

### Chemical similarity network (CSN)

**Figure 1:**
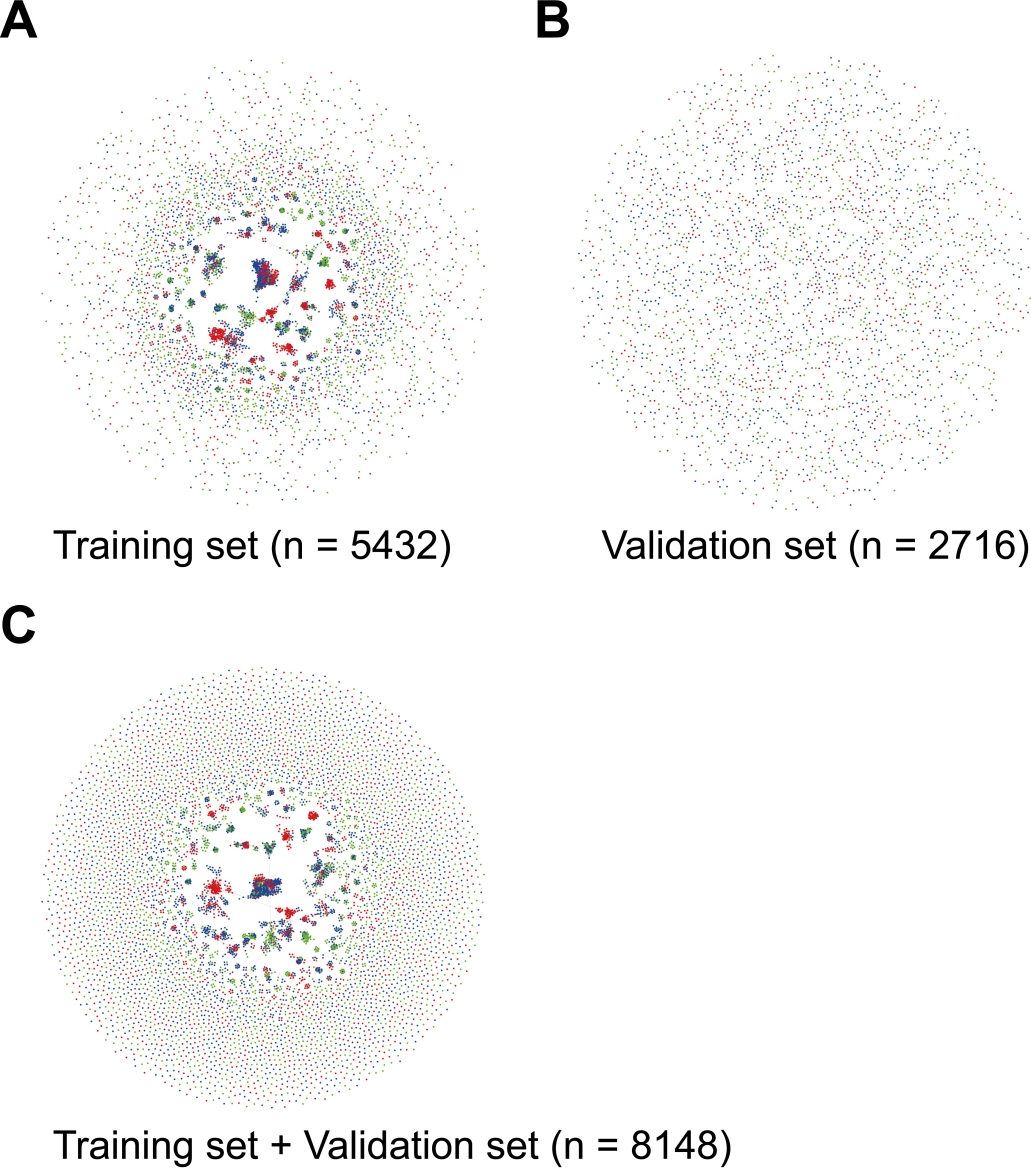
Chemical similarity network illustration of compounds in the training set (A), the validation set (B) and the collection of the two sets (C). Green, blue and red nodes represent P450 non-inhibitors, non-extensive P450 inhibitors, and multi-P450 inhibitors, respectively. Edges indicate ≥80% structural similarity between two compounds.

To investigate the structural consistency of multi-P450 inhibitors, the Tanimoto coefficient was calculated using the chemoinformatics plug-in ChemViz after importing the SMILES strings of the chemicals into Cytoscape v2.8.3 (Smoot et al., 2011). ChemViz is widely used for network visualization of chemicals with similar structures (Wallace et al., 2011; Schlessinger et al., 2012; Su et al., 2012). Herein, a threshold of 0.8 was accepted for Tanimoto coefficient calculation to cluster chemicals with similar structures in a CSN. In the CSN, distinguishably colored nodes represent P450 non-inhibitors, non-extensive P450 inhibitors and multi-P450 inhibitors, and edges indicate ≥80% structural similarity between two chemicals (Fig. 1).

### Molecular descriptor calculation

The 8,148 compounds with complete *in vitro* data were analyzed for structural consistency by building a CSN. Thereafter, they were divided into a training set and a validation set in a 2:1 ratio. All similar compounds were included in the training set, the validation set only contained compounds that were dissimilar to other compounds in both sets. The natural compounds retrieved from TCM Database@Taiwan were incorporated into model application set II. Before molecular descriptor calculation, the natural compounds in model application set II and the literature-reported MBIs were subjected to data preprocessing. Briefly, salts were converted to their corresponding acids or bases, and water molecules were removed from hydrates (Cheng et al., 2011). To avoid potential influences of macromolecules on data overflow of the cascade network model, only the small compounds (molecular weight < 800 Dalton) were considered. All inorganic compounds and noncovalent complexes and mixtures were discarded from our study.

The chemical simulation software Maestro v9.3 (Schrödinger) was used to generate three-dimensional (3D) conformation of all the compounds in each set and export the result as a mol file. The 3D structures of these compounds were generated through LigPrep 2.5a in Schrödinger Suite. After that, the chemoinformatics software PaDEL-Descriptor (Yap, 2011) was applied to calculate the 1D, 2D, and 3D molecular descriptors for each compound in the three sets and the literature-reported MBIs using the mol file containing 3D information as input. In total, 1,875 molecular descriptors were calculated for each compound.

### NNC model building

To identify potential natural multi-P450 inhibitors in model application set II, an NNC model composed of 17 cascaded ANNs was established by constructing a predictive relationship between molecular descriptors and the number of inhibited P450 isoforms. Briefly, all of the molecular descriptors for each of the 8,148 chemicals in the modeling and external validation sets were normalized between 0 and 1, as described previously (Zhu & Kan, 2014). After normalization, a radial basis function (RBF)-ANN with 1-11-1 network architecture was built for each molecular descriptor of the 5,426 chemicals in the modeling set using the Intelligent Problem Solver (IPS) tool in STATISTICA Neural Networks (SNN, Release 4.0E). The normalized molecular descriptor values were used as input variables, with the normalized numbers of inhibited P450 isoforms as output variables. Considering structural diversity, we also established a larger NNC model that consisted of all the chemicals in the modeling and external validation sets. In this study, we named the NNC models NNC model I (*n* = 5,426) and NNC model II (*n* = 8,148), respectively. We followed a step-by-step procedure for NNC model building to set the operating parameters in IPS (File S1).

In this study, the normalized network prediction values were uniformly termed the P450 inhibition score (PIS). Graphpad Prism v6.0 was used to calculate the nonparametric Spearman correlation coefficient (Spearman’s rho) between the PIS values and the normalized numbers of inhibited P450 isoforms. Molecular descriptors containing more chemical structure information related to multi-P450 inhibition have correspondingly higher Spearman’s rho values. After re-sorting in descending order, the molecular descriptors with the highest Spearman’s rho values were highlighted as suitable NCC inputs. Only the molecular descriptors with a Spearman’s rho value > 0.4 were selected to construct NNC models I and II.

Unlike the pyramid-like framework of the NNC model established previously (Zhu & Kan, 2014), a ladder-like architecture was adopted in this study. Briefly, the molecular descriptor with the highest Spearman’s rho was preferentially selected as the starting point for extension of the ladder of ANNs. The remaining molecular descriptors were arranged in turn to build a 2-11-1 network architecture ANN. The ANN was retained only if it resulted in the maximum increase in Spearman’s rho. Thus, this ANN contained two molecular descriptors. With the same operation, the PIS of the ANN was integrated with one of the remaining molecular descriptors in a new ANN with the same network architecture. Similarly, the ANN that contributed to the maximum increase in Spearman’s rho was retained for further extension of the ANN cascade. Such a modeling operation would be terminated artificially until there was no further increase in Spearman’s rho or all of the molecular descriptors were incorporated in NNC model I or II.

### Model validation, comparison, and application

The holdout cross-validation method was applied for internal validation of each ANN in NNC models I and II. IPS divided the modeling set into three subsets (training set, verification set, and testing set) in a 2:1:1 ratio when building each ANN in the NNC model. Thus, one-quarter of all the compounds did not participate in the process of model building but were treated as model testing samples, or internal validation samples. The IPS-given correlation coefficients were compared for the training set (*R*_Tr_) and the testing set (*R*_Te_). The two correlation coefficients measured the linear relationship between the PIS values and the normalized number of inhibited P450 isoforms. Similar *R*_Te_ and *R*_Tr_ in value indicates good generalizability of the corresponding ANN.

To evaluate the overall performance of NNC model I, 2,716 compounds with complete *in vitro* data was used for model validation. For NNC model II, a 10-fold cross-validation method was used as illustrated by Fig. S1. Briefly, the entire compound set (*n* = 8,148) was randomly divided into 10 mutually exclusive groups of nearly equal size. Of these groups nine were selected for model training and the last was used for model validation. The above procedure was repeated 10 times to allow each of the groups to be independently used for validation. Moreover, two regular ANN models were built for model comparison. ANN models I and II used the same compounds and molecular descriptors applied in NNC models I and II, respectively.

Based on the final PIS values obtained from each of the four models, Spearman’s rho was calculated to evaluate whether the PIS values and the number of inhibited P450 isoforms were significantly correlated. Receiver operating characteristic (ROC) curve analysis was performed to evaluate the difference between P450 non-inhibitors (*n* = 0) and P450 inhibitors (*n* = 1–5) and that between non-multi-P450 inhibitors (*n* = 0–2) and multi-P450 inhibitors (*n* = 3–5) using MedCalc v13.0, where *n* refers to the number of inhibited P450 isoforms. Additionally, a Chi-squared test was utilized to investigate the potential impact of structure diversity, model type, and P450 inhibition type on accuracy. Accuracy was calculated as the number of successfully predicted P450 inhibitors and non-inhibitors divided by the sum of all compounds. All the 1,919 compounds in model application set I and all of the natural compounds in model application set II were subjected to the PIS calculation.

### Statistical analysis

Data were expressed as mean ± SEM (standard error of the mean). Statistical analysis was performed with the Spearman correlation test or Chi-squared test using Graphpad Prism v6.0. The methodology of DeLong, DeLong & Clarke-Pearson, (1988) was used for pairwise comparison of ROC curves using MedCalc v13.0. Differences were considered significant at *p* < 0.05.

## Results

The CPID was used to obtain *in vitro* data for non-inhibitors and inhibitors against five P450 isoforms, namely, CYP1A2, CYP2C9, CYP2C19, CYP2D6, and CYP3A4. We calculated 1,875 molecular descriptors for 8,148 small molecules with *in vitro* data to build an NNC-based multi-P450 inhibitor prediction model and subjected it to strict internal and external validation. Structure diversity was considered for model optimization, and NNC models were compared with regular ANN models. Although all of the 1,875 molecular descriptors were initially considered network input without discrimination, only 12 molecular descriptors (Table S4 and Fig. S2) were ultimately selected as inputs in the NNC model based on the most significant correlation between their PIS values and the normalized numbers of inhibited P450 isoforms (Spearman’ rho > 0.4) and the optimal integration effect for elevating Spearman’s rho of the final ANN submodel. After calculating and importing the 12 molecular descriptors for each of the 158,795 natural compounds from the TCM Database@Taiwan, we applied the model to predict natural multi-P450 inhibitors from only 12 molecular descriptors depicting 2D or 3D structural information. Ultimately, ∼22% of the natural compounds were suggested as potential multi-P450 inhibitors by the NNC model established herein. Furthermore, chemical similarity calculation suggested alkaloids as the prevailing parent structures of natural multi-P450 inhibitors.

### Data integration enabling the NNC model to identify multi-P450 inhibition

**Figure 2:**
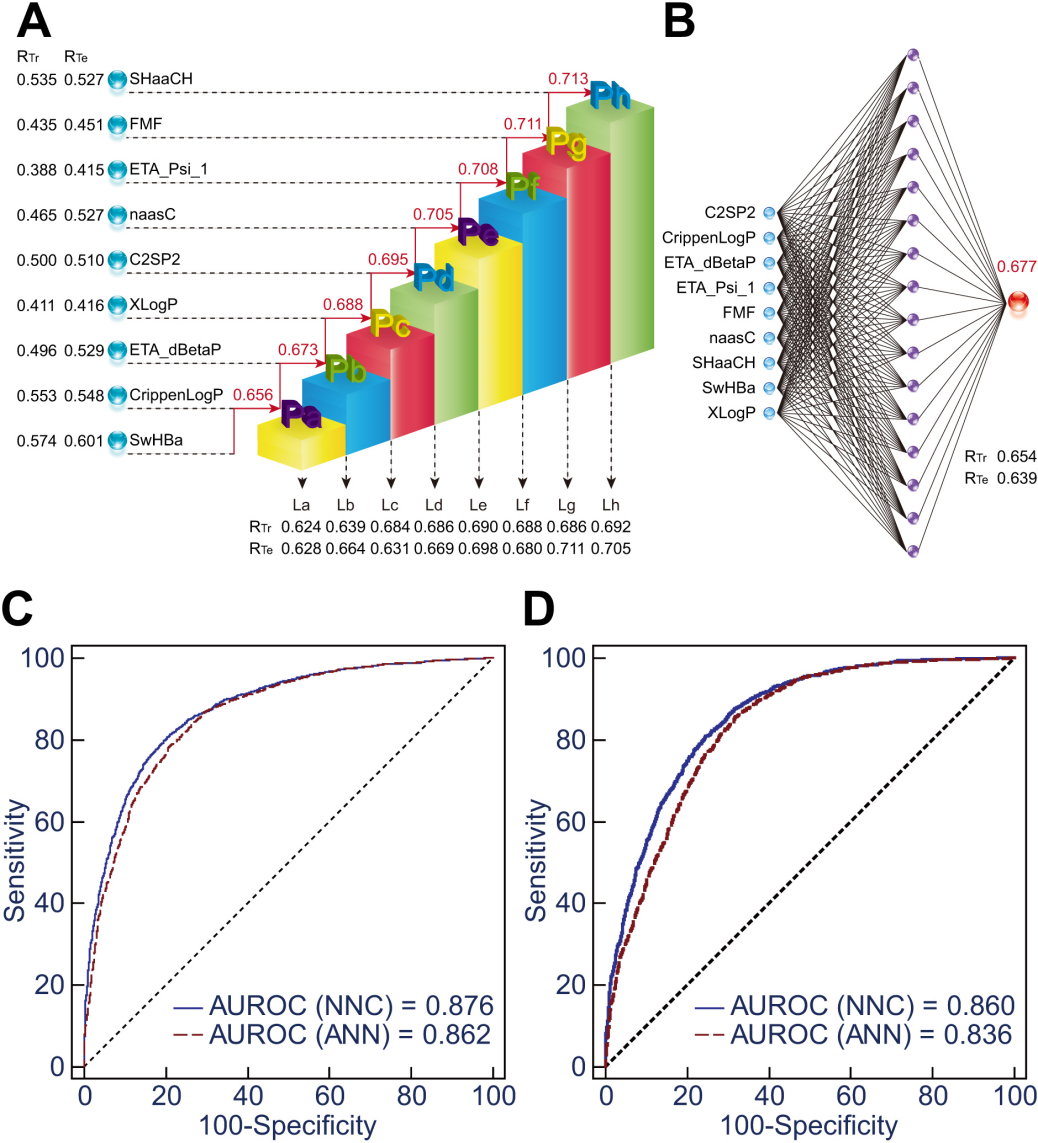
Comparison of NNC model I and ANN model I. (A) Illustration of the framework of NNC model I. La ∾ Lh represent the ladder submodels in which the corresponding molecular descriptors were imported; Pa ∾ Ph are the integrated PIS parameters. For each submodel, the correlation coefficients between the normalized number of inhibited P450 isoforms and P450 inhibition scores of the compounds in the training set (*R*_Tr_) and the testing set (*R*_Te_) are shown. Spearman’s rho for the correlation between the PIS values and the normalized numbers of inhibited P450 isoforms was also calculated for each integrated PIS (top). (B) Illustration of the framework of ANN model I. (C) The AUROCs are 0.876 and 0.862 for discrimination between P450 inhibitors (*n* = 1–5) and P450 non-inhibitors (*n* = 0) using NNC model I and ANN model I, respectively. (D) The AUROCs are 0.860 and 0.836 for identification of non-multi-P450 inhibitors (*n* = 0–2) and multi-P450 inhibitors (*n* = 3–5) using the two models, respectively.

Structure diversity was considered to group compounds used for model training and validation. To evaluate the NNC model architecture based on structure diversity, all similar compounds were classified to the training set, and partial dissimilar compounds were classified to the validation set (Fig. 1). Our results indicate that the PIS of each molecular descriptor included in NNC model I was only weakly correlated with the normalized number of inhibited P450 isoforms, with Spearman’s rho values ranging from 0.413 to 0.620. However, ladder-like data integration by NNC dramatically increased the correlation between chemical structure and multi-P450 inhibition. We verified that the PIS values exported from the final ANN submodel were significantly positively corrected with the normalized number of inhibited P450 isoforms (Spearman’s rho = 0.713, *p* < 0.0001, Fig. 2A). In comparison, ANN model I using the same nine molecular descriptors only contributed a Spearman’s rho of 0.677 (Fig. 2B). Consistent with this, ROC curve analysis indicated a significant increase in the area under the ROC (AUROC) for identifying P450 inhibitors and multi-P450 inhibitors using NNC model I, compared with ANN model I (*p* < 0.0001, Figs. 2C and 2D, and Table S5).

**Figure 3:**
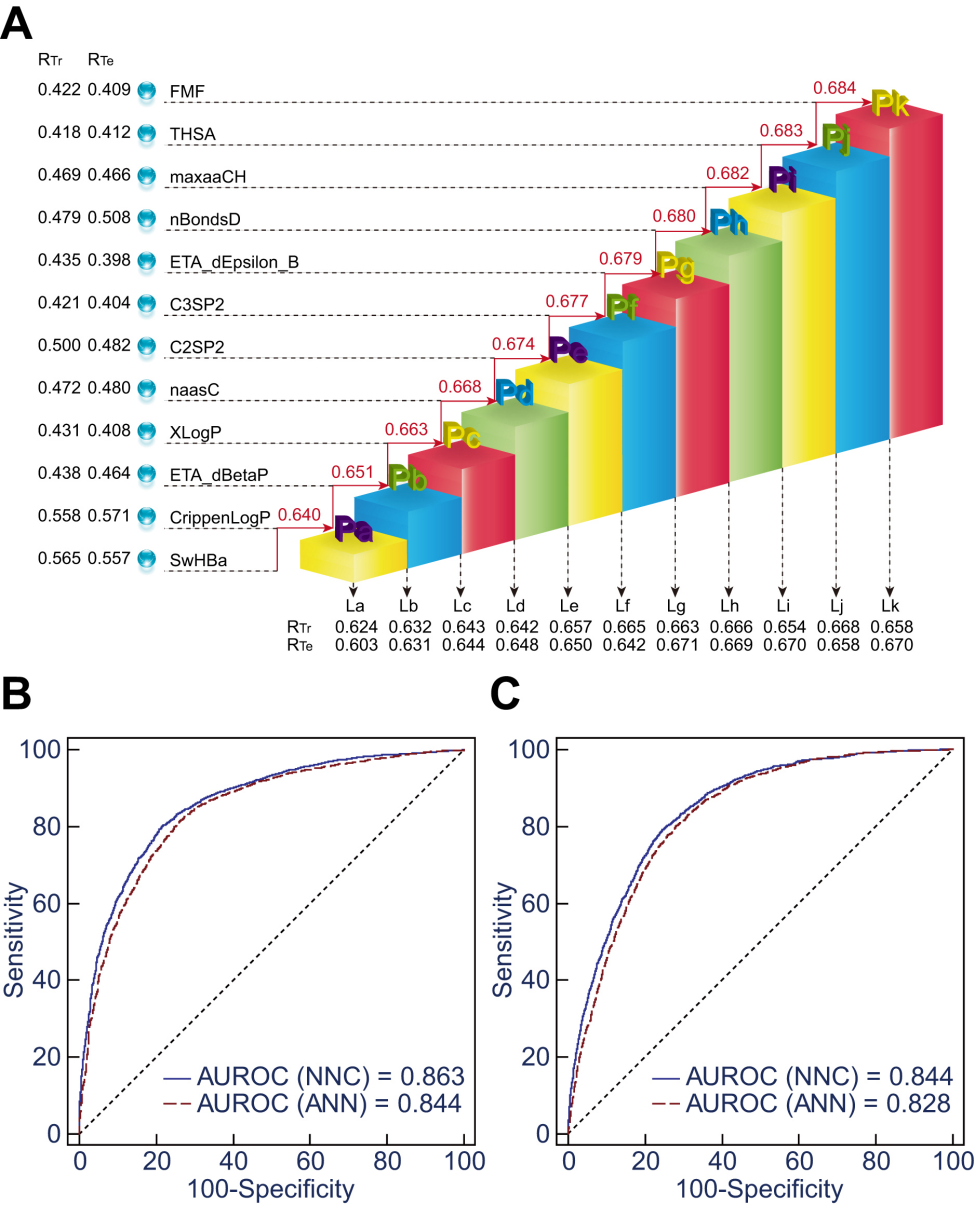
Comparison of NNC model II and ANN model II. (A) Illustration of the framework of NNC model II. La ∾ Lk represent the ladder submodels in which the corresponding molecular descriptors were imported; Pa ∾Pk are the integrated PIS parameter. For each submodel, the correlation coefficients between the normalized number of inhibited P450 isoforms and P450 inhibition scores of the compounds in the training set (*R*_Tr_) and the testing set (*R*_Te_) are shown. Spearman’s rho for the correlation between the PIS values and the normalized numbers of inhibited P450 isoforms was also calculated for each integrated PIS (top). (B) The AUROCs are 0.863 and 0.844 for discrimination between P450 inhibitors (*n* = 1–5) and P450 non-inhibitors (*n* = 0) using NNC model II and ANN model II, respectively. (C) The AUROCs are 0.844 and 0.828 for identification of non-multi-P450 inhibitors (*n* = 0–2) and multi-P450 inhibitors (*n* = 3–5) using the two models, respectively.

We further assessed the predictive power of the two models. We did not observe significant difference in identifying P450 inhibitors (Chi-squared test, *p* = 0.36,Table S6) and multi-P450 inhibitors (Chi-squared test, *p* = 0.44, Table S7) among the 2,716 chemicals in the validation set (Fig. 1B). The global accuracy rates were 78.7% and 77.7% for identifying P450 inhibitors and 76.8% and 75.9% for identifying multi-P450 inhibitors using NNC model I and ANN model I, respectively. However, compared with ANN model I, we found that NNC model I more accurately identified P450 inhibitors in application set I (Chi-squared test, *p* = 0.0018, Table S8). The global accuracy rates were 89.9% and 86.7% for identifying P450 inhibitors using NNC model I and ANN model I, respectively.

All 8,148 compounds in the training and validation sets (Fig. 1C) were used to construct a larger model to enhance the chemical structure diversity of the NCC model architecture. The resulting NNC model II integrated 11 2D molecular descriptors and one 3D molecular descriptor into a single PIS (Fig. 3A). The PIS values exported from the final ANN submodel were significantly positively corrected with the normalized number of inhibited P450 isoforms (Spearman’s rho = 0.684, *p* < 0.0001, Fig. 3A). In comparison, ANN model II using the same 12 molecular descriptors only yielded a Spearman’s rho of 0.652 (*R*_Tr_ = 0.629, *R*_Te_ = 0.625). Consistent with this, ROC curve analysis indicated a significant increase in AUROCs using NNC model II for identifying P450 inhibitors and multi-P450 inhibitors, compared with ANN model II (*p* < 0.0001, Figs. 3B and 3C, and Table S5).

The 1,919 validated P450 inhibitors in application set I were used to compare performance of the two models. Significantly, greater accuracy was observed for NNC model II (Chi-squared test, *p* = 0.036, Table S8). The global accuracy rates were 92.1% and 90.1% for identifying P450 inhibitors using NNC model II and ANN model II, respectively. Chi-squared tests suggested a significant difference in prediction accuracy between NNC models I and II (*p* = 0.021, Table S8) and between ANN models I and II (*p* = 0.001, Table S8). Furthermore, we investigated the potential influence of structural diversity on P450 inhibitor identification by NNC models I and II. Using ChemViz, we found that 281 of the 1,919 P450 inhibitors were structurally similar to the compounds in the training set (Fig. 1A). However, merging the compounds in the training and validation sets increased this number by only 75 to give 356. Chi-squared tests indicate that the percentage of similar compounds significantly decreased from 5.17% (281/5,432) to 4.37% (356/8,148), although the sum of the chemicals for model building increased 33% from 5,432 to 8,148 (*p* = 0.033). This finding implies that structural diversity contributes to higher prediction accuracy for NNC model II than for NNC model I.

### Internal and external validation of the NNC and ANN models

**Figure 4:**
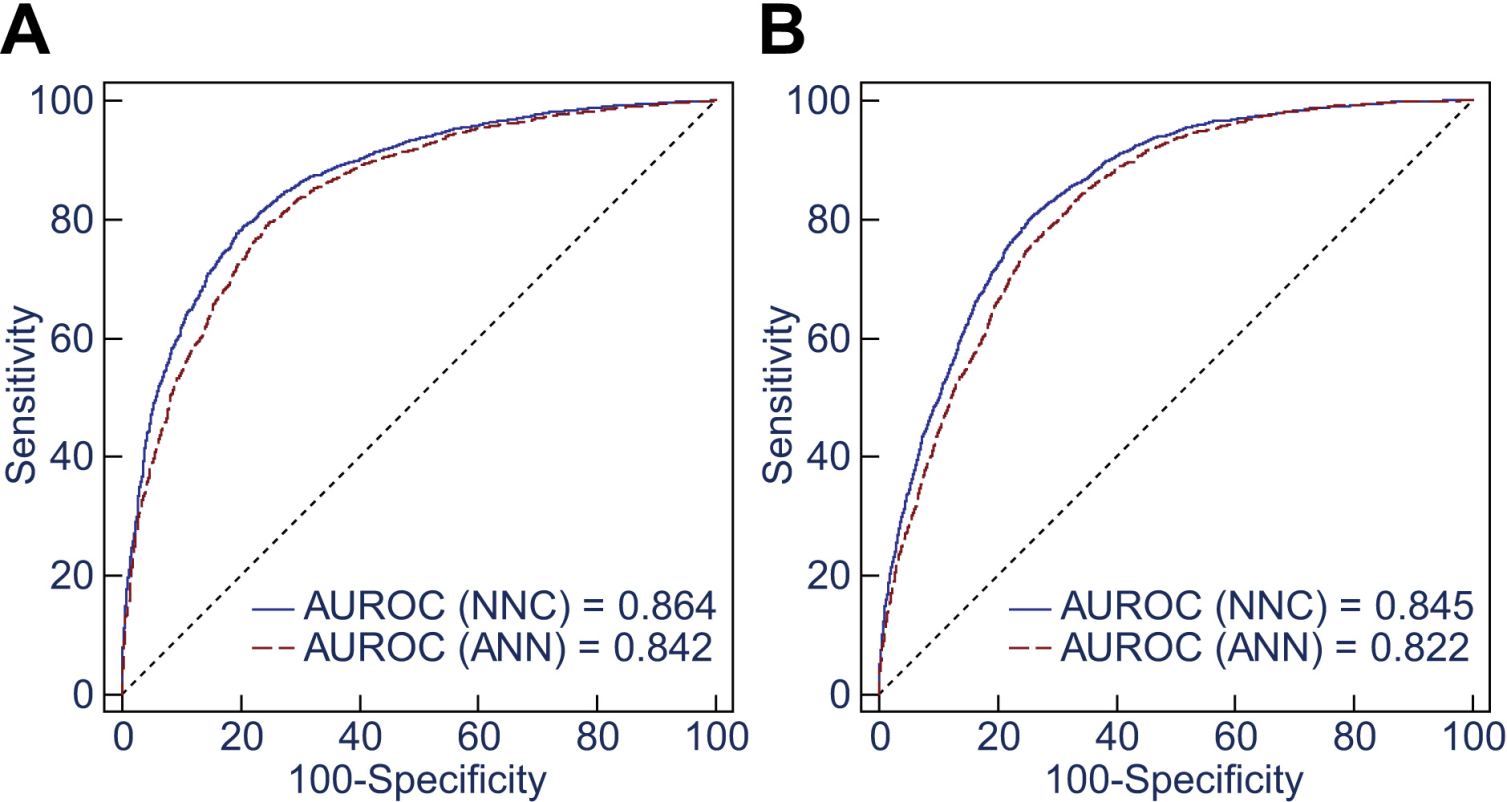
Ten-Fold cross-validation of NNC model II and ANN model II. (A) The AUROCs are 0.864 and 0.842 for discrimination between P450 inhibitors (*n* = 1–5) and P450 non-inhibitors (*n* = 0) using NNC model II and ANN model II, respectively. (B) The AUROCs are 0.845 and 0.822 for identification of non-multi-P450 inhibitors (*n* = 0–2) and multi-P450 inhibitors (*n* = 3–5) using the two models, respectively.

The holdout cross-validation method was used for internal validation of each ANN submodel in the two NNC models and the two ANN models. Similar values of *R*_Te_ and *R*_Tr_ guaranteed satisfactory generalizability of the constructed models (Figs. 2 and 3). A set of 2,716 compounds with complete *in vitro* P450 inhibition data was applied to test NNC model I and ANN model I for method validation. The PIS values exported from the two models were significantly positively corrected with the normalized number of inhibited P450 isoforms (Spearman’s rho = 0.613 and 0.587 for NNC model I and ANN model I, respectively, *p* < 0.0001). For NNC model II and ANN model II, the 10-fold cross-hold method was used for internal validation. Significant correlations between the PIS scores and the normalized number of inhibited P450 isoforms were observed for both models (Spearman’s rho = 0.686 and 0.645 for NNC model II and ANN model II, respectively, *p* < 0.0001), consistent with ROC curve analysis result. NNC model II and ANN model II exhibited good performance for identifying P450 inhibitors and multi-P450 inhibitors (Fig. 4). The global accuracy rates were 81.3% and 80.0% for identifying P450 inhibitors and 78.7% and 77.0% for identifying multi-P450 inhibitors using NNC model II and ANN model II, respectively. Chi-squared tests indicated better performance of NNC model II for identifying P450 inhibitors (*p* = 0.041) and multi-P450 inhibitors (*p* < 0.0001) (Table S9). External validation using 1,919 P450 inhibitors suggested the effectiveness of the above four models (Table S8). In particular, NNC model II showed the highest accuracy of 92.1%. Furthermore, we compared the efficacies of NNC model II and ANN model II in identifying literature-reported MBIs that irreversibly inhibit P450s(Table S10). Although the two models did not show different predictions for the MBIs (Chi-squared test, *p* = 0.41), NNC model II performed better by successfully identifying 126 of the 145 MBIs, whereas ANN model II recognized 121 of the 145 MBIs.

### Application of NNC to identify natural multi-P450 inhibitors

**Figure 5:**
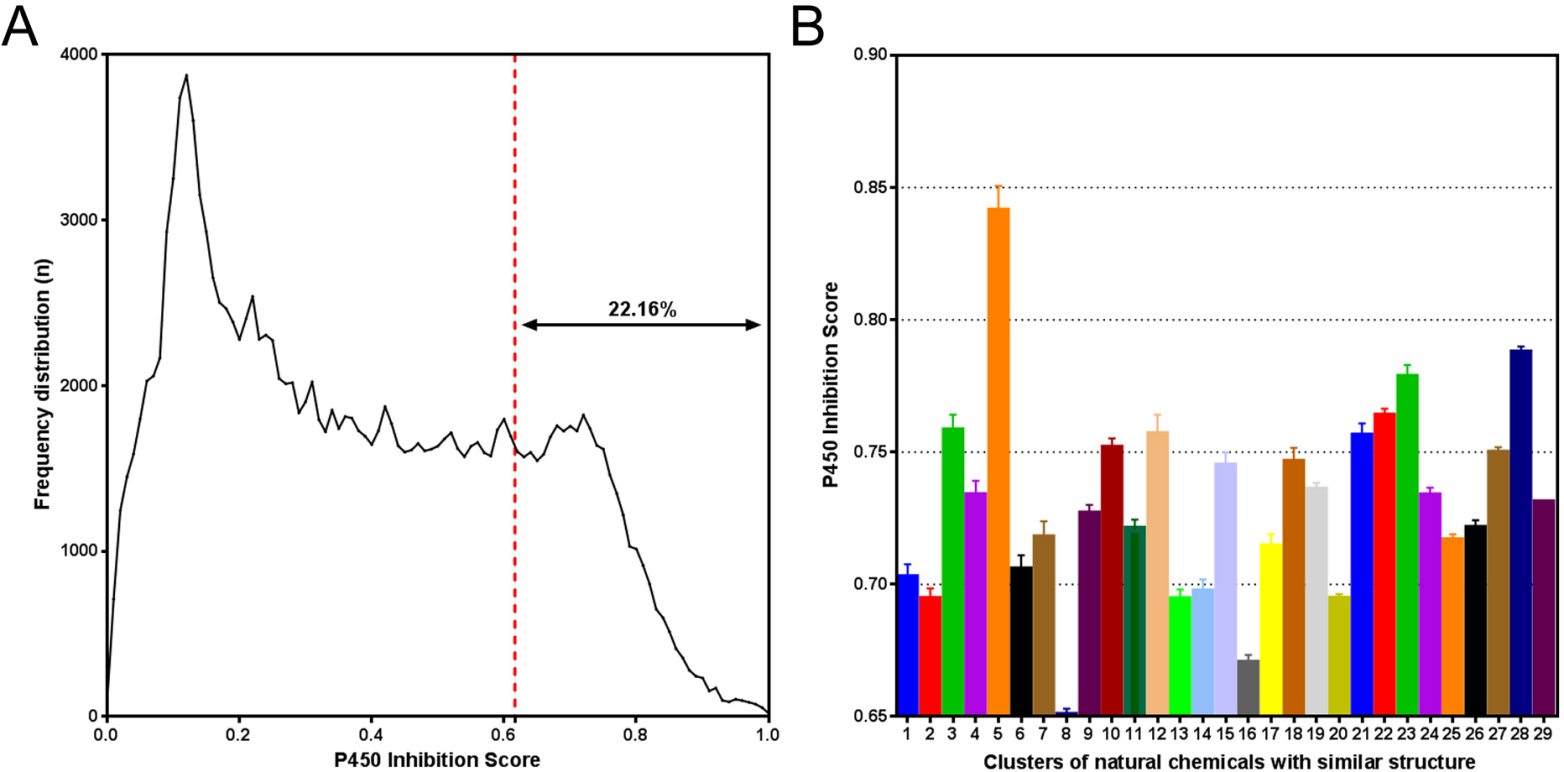
NNC model II identified a high proportion of potential multi-P450 inhibitors among natural compounds. (A) Distribution of PIS for the natural compounds in model application set II. Approximately 22% of natural compounds were identified as potential multi-P450 inhibitors (PIS > 0.6163). (B) Twenty-nine large clusters of compounds with similar structure (*n* > 100) were found for natural compounds with PIS > 0.6163.

A quick view of the whole CPID dataset reveals a large number of compounds without complete *in vitro* inhibition data. For instance, 40.3% of the P450 non-inhibitors in the CPID database lack *in vitro* inhibition data for at least three P450 isoforms. In contrast, only 32.7% of the P450 non-inhibitors possess complete data on *in vitro* inhibition of all five P450 isoforms. This demonstrates widespread inadequacies in experimental validation of thousands of chemicals with respect to inhibition of the main drug-metabolizing P450s. In contrast, such information may be completely missing for the vast majority of natural compounds in the TCM Database@Taiwan. Using NNC model II, we performed an in silico scan of ∼160,000 natural compounds to identify potential multi-P450 inhibitors. The PIS value was calculated for each chemical, and we identified 35,186 potential multi-P450 inhibitors at the optimal ROC threshold (PIS = 0.6163), which accounted for 22.16% of all the natural compounds in the model application set (Fig. 5A). This finding implies the presence of multi-P450 inhibitors among natural compounds. Furthermore, by constructing the CSN of potential multi-P450 inhibitors identified by NNC model II, we identified 29 large compound clusters (*n* > 100), suggesting diverse structural characteristics (Fig. 5B). Identification of a consistent P450 inhibition feature in one cluster raised the accuracy of NNC prediction substantially. Figure S3 presents the 2D structures of the most representative compounds, which possess the largest number of structurally similar neighbors in their individual clusters. The parent structure characteristics suggest that 25 belong to alkaloids.

## Discussion

The effects of multi-P450 inhibition were only recognized recently (Isoherranen et al., 2012). Simultaneously and forcefully blocking multiple metabolic pathways causes an exponential rather than algebraic rise of drug plasma concentrations (Obach & Ryder, 2010), which places patients receiving such combination drug therapy at an enormous risk of excessive drug exposure. Despite extensive application of *in vitro* P450 inhibition assessment of potential drug-like compounds, related methods are mainly focused on investigating the inhibitory potency on individual P450 isoforms, especially for the most important drug-metabolizing P450 enzymes (CYP1A2, CYP2C9, CYP2C19, CYP2D6, and CYP3A4) (Walsky & Boldt, 2008; Nettleton & Einolf, 2011). Even for these five isoforms, a tendency for subjective selection is evident in the performance of P450 inhibition assessments. For instance, within the CPID dataset containing nearly 25,000 unique compounds, inhibition of CYP3A4 was evaluated for >75% of entries, whereas only ∼60% were tested for inhibition of CYP2D6 (Cheng et al., 2011). CYP3A4 may have more available *in vitro* inhibition data because its activity is known to be more vulnerable to chemical effects, possibly because it possesses a larger binding cavity than CYP2D6 (Tickle & Jhoti, 2004; Williams et al., 2012; Wang et al., 2012). Thus, CYP3A4 has been the focus of much DDI research.

Notably, incomprehensive data limit determination of serious adverse DDIs due to multi-P450 inhibition. Additionally, as experimental data are incomplete for many compounds, we cannot confidently assess the proportion of multi-P450 inhibitors or whether simultaneous inhibition of multiple P450s is significant. Until now, appropriate and simple methods to estimate the consequence of multi-P450 inhibition on the alteration of blood drug concentration have not been available (Obach & Ryder, 2010). This prevents experimental evaluation of the significance of multi-P450 inhibition.

The successes achieved by previous studies toward establishing virtual P450 inhibition models (Molnár & KeserűG, 2002; Jensen et al., 2007) prompted us to attempt a similar approach to comprehensively scan the structure–activity property ‘multi-P450 inhibition’ in the CPID database of nearly 25,000 unique compounds. Predictions from our NNC model reveal that multi-P450 inhibition may be a widespread occurrence in numerous compounds with diverse chemical structures. This finding suggests that comprehensive *in vitro* inhibition assessment of drug metabolism-related P450s should be seriously considered for potential drug-like compounds in new drug development.

As overlapping structural information was commonly found among molecular descriptors calculated by PaDEL-Descriptor, only 11 2D and one 3D molecular descriptors were included as inputs in NNC model II. Despite the small number of molecular descriptors used, a correlation coefficient of 0.684 between PIS and the number of inhibited P450s clearly demonstrates that the NNC model is suitable for predicting multi-P450 inhibitors. We propose that the ladder-like network organization strengthened the prediction effectiveness of the NNC model, in which 11 RBF-ANN submodels were sequentially cascaded to allow the flow and convergence of information originating from different molecular descriptors. The results of internal and external validation suggest good generalizability of each ANN unit and guarantee the overall consistent performance of the NNC model in multi-P450 inhibitor identification. Compared with ANN model II, our results highlight the predictive advantage of the NNC model ANN architecture using the same molecular descriptors as inputs. This is consistent with our previous study, in which we validated the superior prediction performance of the NNC model ANN architecture to the ANN model (Zhu & Kan, 2014). Furthermore, our findings imply that enriching the structure diversity of compounds in NNC model contributes to more accurate prediction.

The optimized NNC model built herein, NNC model II, provided a novel opportunity for rapid, high-throughput screening of the multi-P450 inhibition potential for natural compounds to explore whether natural multi-P450 inhibitors exist, how common they are, and whether they share common structural features. NNC model II identified 35,186 potential multi-P450 inhibitors from 158,795 unique natural compounds. This finding suggests that possible multi-P450 inhibition by natural chemicals may not be a rare event and should be considered when *in vitro* assessment is performed. Furthermore, CSN building verified this finding as isolated chemical clusters imply diverse rather than consistent parent structures of potential natural multi-P450 inhibitors. This prediction was consistent with current knowledge about naturally occurring chemical-caused P450 enzyme inhibition (Delgoda & Westlake, 2004). The present method indicated that compared with other classes of natural compounds, alkaloids may have increased potential for multi-P450 inhibition. This finding was supported by previous experimental studies. For example, isoquinoline alkaloids, such as compounds 24 and 27 (Fig. S3), were identified by our method to be potential multi-P450 inhibitors. This result was consistent with a study from Salminen et al. (2011), in which isoquinoline alkaloids were shown to remarkably inhibit the enzyme activity of multiple P450s.

In conclusion, we established a feasible method for virtually screening the potential for multi-P450 inhibition in compounds with known chemical structures, and application of our model revealed a prevalence of multi-P450s inhibition by natural compounds, especially alkaloids. This finding suggests that serious caution should be observed when alkaloid extract or traditional medicines rich in such substances are used in combination with prescription medicines mainly metabolized by P450s *in vivo*. Our models were constructed using only the chemical structure information of compounds. Thus, more new inputs reflecting multi-P450 inhibition should be investigated and considered for inclusion in the NNC model in future studies, and ligand-protein docking simulations should be performed to determine P450 inhibition-related docking simulation parameters, as suggested by several previous studies (Shi et al., 2011; VandenBrink et al., 2012; Zhou et al., 2013; Shityakov et al., 2014). Furthemore, examination of these predictions by *in vitro* or *in vivo* experimental methods is necessary, especially for the natural compounds we identified with high likelihoods of multi-P450 inhibition. Nevertheless, our results suggest the superior predictive power of NNC model architecture to regular ANN model. Such a novel model architecture can be used for other research fields of quantitative structure–activity relationship.

## Supplemental Information

10.7717/peerj.1524/supp-1 File S1 Step-by-step procedure for constructing NNC model.

10.7717/peerj.1524/supp-2 Figure S1 Illustration of the 10-fold cross-validation method

10.7717/peerj.1524/supp-3 Figure S2 Distribution of the 12 molecular descriptors used in NNC model II

10.7717/peerj.1524/supp-4 Figure S3 Representative natural multi-P450 inhibitors identified by NNC model II. Chemicals in clusters of 1 8˜, 10 2˜2, 24, 25, 27, and 28 are classified as alkaloids

10.7717/peerj.1524/supp-5 Table S1 A list of the compounds in the training and validation sets

10.7717/peerj.1524/supp-6 Table S2 Description of the training and validation sets in the term of P450 inhibition

10.7717/peerj.1524/supp-7 Table S3 A list of the compounds in the model application set I

10.7717/peerj.1524/supp-8 Table S4 Description of the 12 molecular descriptors used in NNC model II

10.7717/peerj.1524/supp-9 Table S5 Performance of four models in identifying P450 inhibitors and multi-P450 inhibitors

10.7717/peerj.1524/supp-10 Table S6 Comparison of ANN model I and NNC model I in identifying P450 inhibitors in the validation set

10.7717/peerj.1524/supp-11 Table S7 Comparison of ANN model I and NNC model I in identifying multi-P450 inhibitors in the validation set

10.7717/peerj.1524/supp-12 Table S8 Pairwise comparison of the four models in identifying P450 inhibitors in the model application set I

10.7717/peerj.1524/supp-13 Table S9 Comparison of ANN model II and NNC model II in identifying P450 inhibitors and multi-P450 inhibitors

10.7717/peerj.1524/supp-14 Table S10 Prediction result for literature-reported MBIs using NNC model II

## Additional Information and Declarations

### Data Availability

The following information was supplied regarding data availability: The research in this article did not generate any raw data.

## References

[ref-1] Ajayi FO, Sun H, Perry J (2000). Adverse drug reactions: a review of relevant factors. Journal of Clinical Pharmacology.

[ref-2] Chen CY (2011). TCM Database@Taiwan: the world’s largest traditional Chinese medicine
database for drug screening in silico. PLoS ONE.

[ref-3] Cheng F, Yu Y, Shen J, Yang L, Li W, Liu G, Lee PW, Tang Y (2011). Classification of cytochrome P450 inhibitors and noninhibitors using
combined classifiers. Journal of Chemical Information and Modeling.

[ref-4] Delgoda R, Westlake AC (2004). Herbal interactions involving cytochrome P450 enzymes: a mini
review. Toxicological Reviews.

[ref-5] Guengerich FP (2008). Cytochrome P450 and chemical toxicology. Chemical Research in Toxicology.

[ref-6] DeLong ER, DeLong DM, Clarke-Pearson DL (1988). Comparing the areas under two or more correlated receiver operating
characteristic curves: a nonparametric approach. Biometrics.

[ref-7] Irwin JJ, Sterling T, Mysinger MM, Bolstad ES, Coleman RG (2012). ZINC: a free tool to discover chemistry for biology. Journal of Chemical Information and Modeling.

[ref-8] Isoherranen N, Lutz JD, Chung SP, Hachad H, Levy RH, Ragueneau-Majlessi I (2012). Importance of multi-P450 inhibition in drug-drug interactions: evaluation
of incidence, inhibition magnitude, and prediction from *in vitro*
data. Chemical Research in Toxicology.

[ref-9] Jensen BF, Vind C, Padkjaer SB, Brockhoff PB, Refsgaard HH (2007). In silico prediction of cytochrome P450 2D6 and 3A4 inhibition using
Gaussian kernel weighted k-nearest neighbor and extended connectivity fingerprints,
including structural fragment analysis of inhibitors versus
noninhibitors. Journal of Meidcal Chemistry.

[ref-10] Lazarou J, Pomeranz BH, Corey PN (1998). Incidence of adverse drug reactions in hospitalized patients: a
meta-analysis of prospective studies. Jama the Journal of the American Medical Association.

[ref-11] Lin JH, Lu AY (1998). Inhibition and induction of cytochrome P450 and the clinical
implications. Clinical Pharmacokinetics.

[ref-12] Mishra NK (2011). Computational modeling of P450s for toxicity prediction. Expert Opinion on Drug Metabolism & Toxicology.

[ref-13] Molnár L, KeserűG M (2002). A neural network based virtual screening of cytochrome P450 3A4
inhibitors. Bioorganic & Medicinal Chemistry Letters.

[ref-14] Nettleton DO, Einolf HJ (2011). Assessment of cytochrome P450 enzyme inhibition and inactivation in drug
discovery and development. Current Topics in Medicinal Chemistry.

[ref-15] Obach RS, Ryder TF (2010). Metabolism of ramelteon in human liver microsomes and correlation with the
effect of fluvoxamine on ramelteon pharmacokinetics. Drug Metabolism and Disposition.

[ref-16] Pelkonen O, Turpeinen M, Hakkola J, Honkakoski P, Hukkanen J, Raunio H (2008). Inhibition and induction of human cytochrome P450 enzymes: current
status. Archives of Toxicology.

[ref-17] Rendic S, Di Carlo FJ (1997). Human cytochrome P450 enzymes: a status report summarizing their reactions,
substrates, inducers, and inhibitors. Drug Metabolism Reviews.

[ref-18] Salminen KA, Meyer A, Jerabkova L, Korhonen LE, Rahnasto M, Juvonen RO, Imming P, Raunio H (2011). Inhibition of human drug metabolizing cytochrome P450 enzymes by plant
isoquinoline alkaloids. Phytomedicine.

[ref-19] Schlessinger A, Wittwer MB, Dahlin A, Khuri N, Bonomi M, Fan H, Giacomini KM, Sali A (2012). High selectivity of the *γ*-aminobutyric acid transporter 2
(GAT-2, SLC6A13) revealed by structure-based approach. Journal of Biological Chemistry.

[ref-20] Shi R, Li J, Cao X, Zhu X, Lu X (2011). Exploration of the binding of proton pump inhibitors to human P450 2C9
based on docking and molecular dynamics simulation. Journal of Molecular Modeling.

[ref-21] Shityakov S, Puskás I, Roewer N, Förster C, Broscheit J (2014). Three-dimensional quantitative structure–activity relationship and docking
studies in a series of anthocyanin derivatives as cytochrome P450 3A4
inhibitors. Advances & Applications in Bioinformatics & Chemistry.

[ref-22] Smoot ME, Ono K, Ruscheinski J, Wang PL, Ideker T (2011). Cytoscape 2.8: new features for data integration and network
visualization. Bioinformatics.

[ref-23] Spaggiari D, Geiser L, Daali Y, Rudaz S (2014). A cocktail approach for assessing the *in vitro* activity of
human cytochrome P450s: an overview of current methodologies. Journal of Pharmaceutical & Biomedical Analysis.

[ref-24] Su Z, Zhang B, Zhu W, Du Z (2012). In silico and *in vivo* evaluation of flavonoid extracts on
CYP2D6-mediated herb-drug interaction. Journal of Molecular Modeling.

[ref-25] Tanaka E (1998). Clinically important pharmacokinetic drug-drug interactions: role of
cytochrome P450 enzymes. Journal of Clinical Pharmacy & Therapeutics.

[ref-26] Tickle IJ, Jhoti H (2004). Crystal structures of human cytochrome P450 3A4 bound to metyrapone and
progesterone. Science.

[ref-27] VandenBrink BM, Foti RS, Rock DA, Wienkers LC, Wahlstrom JL (2012). Prediction of CYP2D6 drug interactions from *in vitro* data:
evidence for substrate-dependent inhibition. Drug Metabolism and Disposition.

[ref-28] Veith H, Southall N, Huang R, James T, Fayne D, Artemenko N, Shen M, Inglese J, Austin CP, Lloyd DG, Auld DS (2009). Comprehensive characterization of cytochrome P450 isozyme selectivity
across chemical libraries. Nature Biotechnology.

[ref-29] Wallace IM, Bader GD, Giaever G, Nislow C (2011). Displaying chemical information on a biological network using
Cytoscape. Methods in Molecular Biology.

[ref-30] Walsky RL, Boldt SE (2008). *In vitro* cytochrome P450 inhibition and
induction. Current Drug Metabolism.

[ref-31] Wang A, Savas U, Hsu MH, Stout CD, Johnson EF (2012). Crystal structure of human cytochrome P450 2D6 with prinomastat
bound. Journal of Biological Chemistry.

[ref-32] Wang Y, Xiao J, Suzek TO, Zhang J, Wang J, Bryant SH (2009). PubChem: a public information system for analyzing bioactivities of small
molecules. Nucleic Acids Research.

[ref-33] Williams JA, Hyland R, Jones BC, Smith DA, Hurst S, Goosen TC, Peterkin V, Koup JR, Ball SE (2004). Drug-drug interactions for UDP-glucuronosyltransferase substrates: a
pharmacokinetic explanation for typically observed low exposure (AUCi/AUC)
ratios. Drug Metabolism and Disposition.

[ref-34] Williams PA, Cosme J, Vinkovic DM, Ward A, Angove HC, Day PJ, Vonrhein C, Wang A, Savas U, Hsu MH, Stout CD, Johnson EF (2012). Crystal structure of human cytochrome P450 2D6 with prinomastat
bound. Journal of Biological Chemistry.

[ref-35] Yap CW (2011). PaDEL-descriptor: an open source software to calculate molecular
descriptors and fingerprints. Journal of Computational Chemistry.

[ref-36] Zhou S, Gao Y, Jiang W, Huang M, Xu A, Paxton JW (2003). Interactions of herbs with cytochrome P450. Drug Metabolism Reviews.

[ref-37] Zhou X, Wang Y, Hu T, Or PM, Wong J, Kwan YW, Wan DC, Hoi PM, Lai PB, Yeung JH (2013). Enzyme kinetic and molecular docking studies for the inhibitions of
miltirone on major human cytochrome P450 isozymes. Phytomedicine.

[ref-38] Zhu W, Kan X (2014). Network cascade optimizes microRNA biomarker selection for nasopharyngeal
cancer prognosis. PLoS ONE.

