# Supplementary material for: Artificial neural network cascade identifies multi-P450 inhibitors in natural compounds
Source: PeerJ. 2015 Dec 21;3:e1524. doi: 10.7717/peerj.1524 (PMC4696407; doi:10.7717/peerj.1524)
Supplement: File S1 [file peerj-03-1524-s001.docx]

**Step-by-step procedure for constructing NNC model**

**Step 1:** The software STATISTICA Neural Networks (SNN, Release 4.0E) was used to screen each of the 1875 molecular descriptors calculated by PaDEL-Descriptor, one by one. After SNN was started, a dataset should be firstly created in the Data Set Editor by importing single normalized molecular descriptor as input and the corresponding normalized number of inhibited P450 isoforms (0 isoform: 0; 1 isoform: 0.2; 2 isoforms: 0.4; 3 isoforms: 0.6; 4 isoforms: 0.8; 5 isoforms: 1.0) as output. The number of the rows in the dataset should be correspondingly expended by adding cases to ensure that it can exactly contain all the values of the normalized molecular descriptor and normalized number of inhibited P450 isoforms of all the model-building molecules.

**Step 2:** Once the dataset was successfully filled, the Intelligent Problem Solver (IPS) tool should be activated for searching and building an ANN for the given molecular descriptor. The version “Advanced” and the problem type “Standard” should be sequentially selected, and Radial basis function (RBF) was then selected as network type. It should be especially noted that the opinion “determine network complexity automatically” be removed to make the number of hidden units of the ANN fixed at 11. Adoption of the “Throughout” duration of design process allowed the IPS tool more opportunities to find a RBF network with good performance as much as possible. In fact, only the network with the best performance would be retained by setting a strict limitation of “maximum number of network” as 1.

**Step 3:** The ANN model outputs were normalized to digitals from 0 to 1 that were named as P450 inhibition score (PIS). And then, nonparametric Spearman correlation test was performed to assess whether the PIS values be significantly correlated with the normalized number of inhibited P450 isoforms (Spearman’s rho > 0.4, *p* < 0.05). If Spearman’s rho exceeded this threshold, the IPS network searching result should be saved into a .sta file named as the name of the molecular descriptor for further NNC model building. The statistics software Graphpad Prism v6.0 was used to perform the nonparametric Spearman correlation test. Spearman’s rho was calculated as:

$$Spearman's rho=1-\frac{6\sum d_{i}^{2}}{n(n^{2}-1)}$$

where n is the sum of molecules and di is the difference between ranks x_i_ and y_i_, which are converted from the raw scores X_i_ and Y_i_. X*i* is the PIS value of the molecule *i* and Y*i* the normalized number of P450 isoforms inhibited by the molecule *i*.

**Step 4:** Significant molecular descriptors (Spearman’s rho > 0.4, *p* < 0.05) were used to build a NNC model. The molecular descriptor with the highest Spearman’s rho was applied as the starting point of the ladder of the NNC model. After that, the other molecular descriptors were selected one by one to be combined with it for building an ANN with 2-12-1 network architecture by using the IPS tool. The normalized values of the two molecular descriptors were used as inputs and the normalized number of inhibited P450 isoforms used as output. After that, the IPS tool was run for network searching and building as described in Step 2. As the opinion “determine network complexity automatically” was removed, the number of hidden units of the ANN was fixed at 12. Graphpad Prism v6.0 was used to calculate Spearman’s rho between the normalized network output ( or PIS) and the normalized number of P450 isoforms as described in Step 3. If a molecular descriptor contributed to the largest increase in Spearman’s rho, compared with the Spearman’s rho of the starting molecular descriptor, the ANN network composed of it and the starting molecular descriptor should be saved as the first NNC submodel.

**Step 5:** The first NNC submodel contained two molecular descriptors, which was then used as a new starting point to extend the ladder of the NNC model. The remaining molecular descriptors were selected one by one to be combined with the PIS of the NNC submodel for building an ANN with 2-12-1 network architecture. Graphpad Prism v6.0 was used to calculate Spearman’s rho between the normalized network outputs of the ANN built and the normalized number of P450 isoforms. If compared with the other molecular descriptors a molecular descriptor resulting in the largest increase in Spearman’ rho, the ANN network composed of it and the PIS of the first NNC submodel should be saved as the second submodel in the NNC model.

**Step 6:** The above procedure as described in Step 5 should be repeated until there was no molecular descriptor available for building new submodel that further extend the ladder of the NNC model or no obvious increase in Spearman’s rho was obtained by integrating any of the remaining molecular descriptors with the PIS of the NNC submodel at the end of the ladder of the NNC model (Increment of Spearman’s rho < 0.001).
