# Supplementary figures and images for: Artificial neural network cascade identifies multi-P450 inhibitors in natural compounds
Source: PeerJ. 2015 Dec 21;3:e1524. doi: 10.7717/peerj.1524 (PMC4696407; doi:10.7717/peerj.1524)

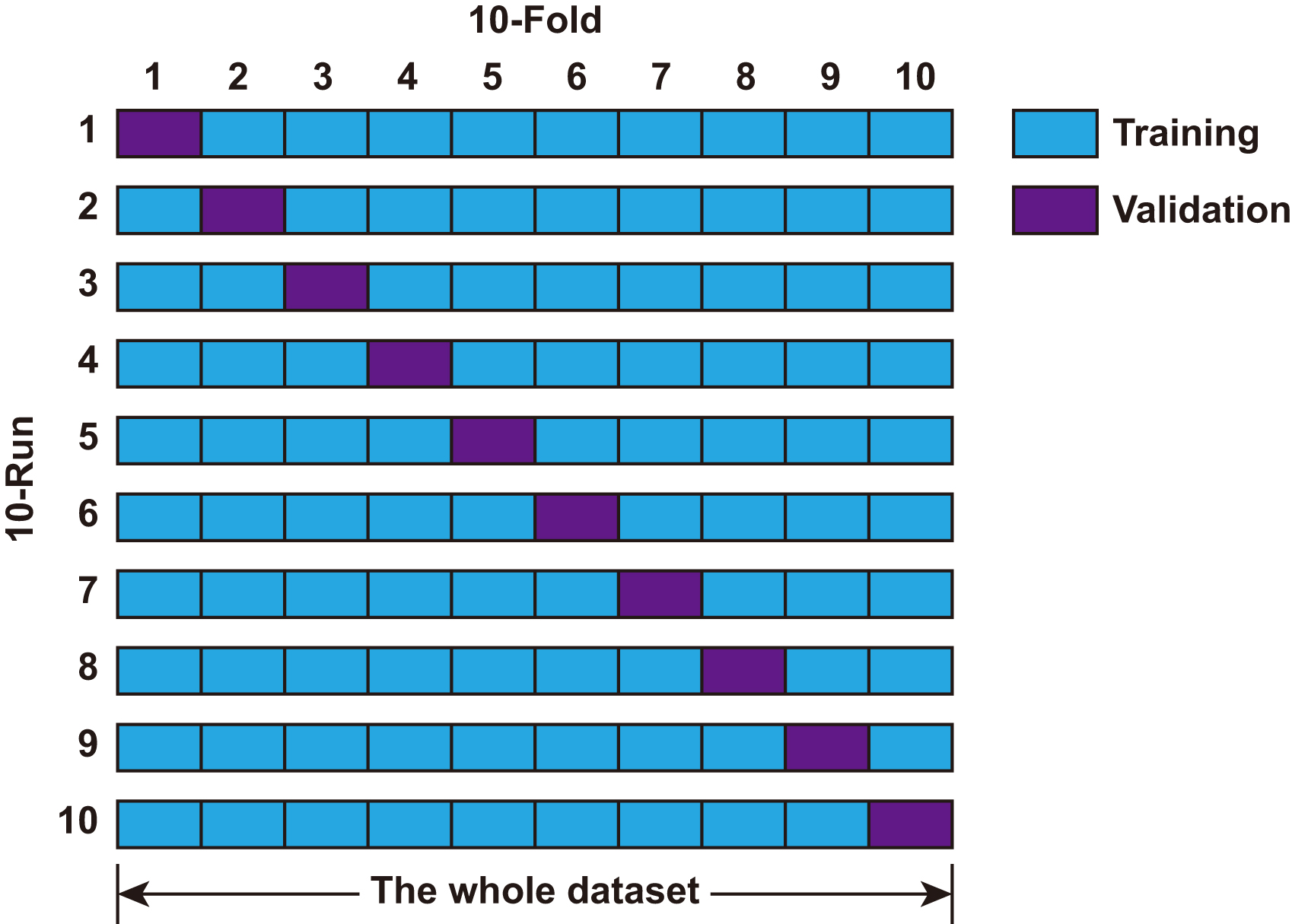

Supplement: Figure S1 [file peerj-03-1524-s002.jpg]

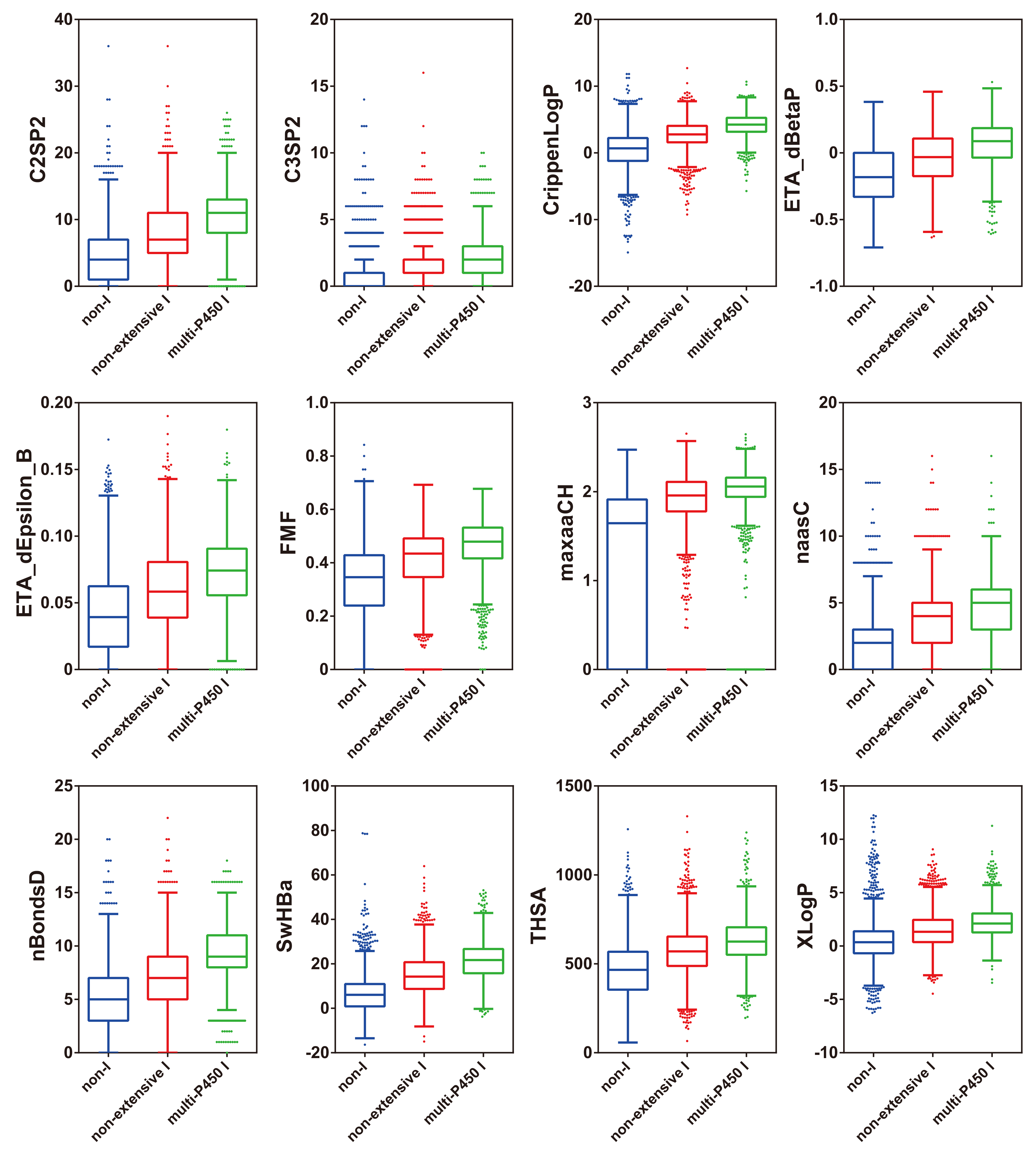

Supplement: Figure S2 [file peerj-03-1524-s003.jpg]

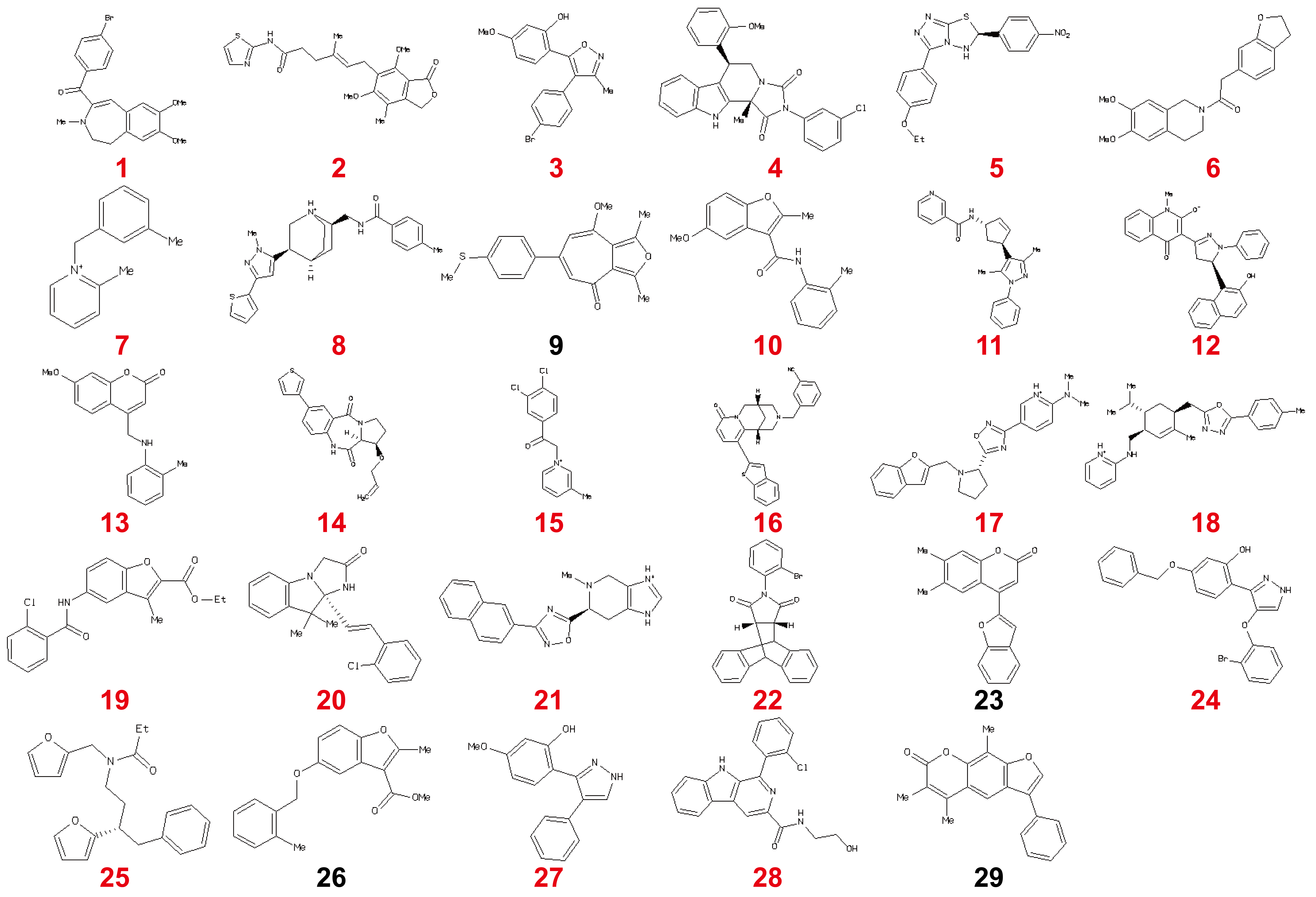

Supplement: Figure S3 [file peerj-03-1524-s004.jpg]
