# Supplementary material for: Artificial neural network cascade identifies multi-P450 inhibitors in natural compounds
Source: PeerJ. 2015 Dec 21;3:e1524. doi: 10.7717/peerj.1524 (PMC4696407; doi:10.7717/peerj.1524)
Supplement: Table S2 [file peerj-03-1524-s006.docx]

**Table S2.** Description of the training and validation sets in the term of P450 inhibition.

| dataset | P450 non-inhibitor (n) | non-extensive P450 inhibitor (n) | multi-P450 inhibitor (n) |
| --- | --- | --- | --- |
| training set | 1681 | 2188 | 1563 |
| validation set | 841 | 1095 | 780 |
