# Supplementary material for: Artificial neural network cascade identifies multi-P450 inhibitors in natural compounds
Source: PeerJ. 2015 Dec 21;3:e1524. doi: 10.7717/peerj.1524 (PMC4696407; doi:10.7717/peerj.1524)
Supplement: Table S4 [file peerj-03-1524-s008.docx]

**Table S4.** Description of the 12 molecular descriptors used in NNC model II.

| Molecular descriptor | Description | Class |
| --- | --- | --- |
| C2SP2 | Doubly bound carbon bound to two other carbons | 2D |
| C3SP2 | Doubly bound carbon bound to three other carbons | 2D |
| CrippenLogP | Crippen's LogP | 2D |
| ETA_dBetaP | A measure of relative unsaturation content relative to molecular size | 2D |
| ETA_dEpsilon_B | A measure of contribution of unsaturation | 2D |
| FMF | Complexity of a molecule | 2D |
| maxaaCH | Maximum atom-type E-State: :CH: | 2D |
| naasC | Count of atom-type E-State: :C:- | 2D |
| nBondsD | Number of double bonds | 2D |
| SwHBa | Sum of E-States for weak hydrogen bond acceptors | 2D |
| THSA | Sum of solvent accessible surface areas of atoms with absolute value of partial charges less than 0.2 | 3D |
| XLogP | XLogP | 2D |
