# Supplementary material for: Artificial neural network cascade identifies multi-P450 inhibitors in natural compounds
Source: PeerJ. 2015 Dec 21;3:e1524. doi: 10.7717/peerj.1524 (PMC4696407; doi:10.7717/peerj.1524)
Supplement: Table S5 [file peerj-03-1524-s009.docx]

**Table S5.** Performance of four models in identifying P450 inhibitors and multi-P450 inhibitors.

| model | Spearman’s rho | AUROC (P450 inhibition) | AUROC (multi-P450 inhibition) |
| --- | --- | --- | --- |
| ANN I | 0.677 | 0.862 | 0.836 |
| NNC I | 0.713 | 0.876 | 0.860 |
| ANN II | 0.652 | 0.844 | 0.828 |
| NNC II | 0.684 | 0.863 | 0.844 |

ANN: artificial neural network; NNC: neural network cascade; AUROC: area under the ROC curve.
