# Supplementary material for: Artificial neural network cascade identifies multi-P450 inhibitors in natural compounds
Source: PeerJ. 2015 Dec 21;3:e1524. doi: 10.7717/peerj.1524 (PMC4696407; doi:10.7717/peerj.1524)
Supplement: Table S6 [file peerj-03-1524-s010.docx]

**Table S6.** Comparison of ANN model I and NNC model I in identifying P450 inhibitors in the validation set (n = 2716).

| model | compound (n) | successfully predicted (n) | accuracy | *p* (Chi-squared test) |
| --- | --- | --- | --- | --- |
| ANN I | 2716 | 2109 | 77.7% | 0.36 |
| NNC I | 2716 | 2137 | 78.7% |  |

ANN: artificial neural network; NNC: neural network cascade; successfully predicted (n): the total number of compounds that were successfully predicted in term of P450 inhibition.
