# Supplementary material for: Artificial neural network cascade identifies multi-P450 inhibitors in natural compounds
Source: PeerJ. 2015 Dec 21;3:e1524. doi: 10.7717/peerj.1524 (PMC4696407; doi:10.7717/peerj.1524)
Supplement: Table S7 [file peerj-03-1524-s011.docx]

**Table S7.** Comparison of ANN model I and NNC model I in identifying multi-P450 inhibitors in the validation set (n = 2716).

| model | compounds (n) | successfully predicted (n) | accuracy | *p* (Chi-squared test) |
| --- | --- | --- | --- | --- |
| ANN I | 2716 | 2062 | 75.9% | 0.44 |
| NNC I | 2716 | 2086 | 76.8% |  |

ANN: artificial neural network; NNC: neural network cascade; successfully predicted (n): the total number of chemicals that were successfully predicted in term of multi-P450 inhibition.
