# Supplementary material for: Artificial neural network cascade identifies multi-P450 inhibitors in natural compounds
Source: PeerJ. 2015 Dec 21;3:e1524. doi: 10.7717/peerj.1524 (PMC4696407; doi:10.7717/peerj.1524)
Supplement: Table S8 [file peerj-03-1524-s012.docx]

**Table S8.** Pairwise comparison of the four models in identifying P450 inhibitors in the model application set I (n = 1919).

| model | compounds (n) | successfully predicted (n) | accuracy | *p* (Chi-squared test) |
| --- | --- | --- | --- | --- |
| ANN I ~ NNC I |  |  |  |  |
| ANN I | 1919 | 1664 | 86.7% | 0.0018 |
| NNC I | 1919 | 1726 | 89.9% |  |
| ANN II ~ NNC II |  |  |  |  |
| ANN II | 1919 | 1729 | 90.1% | 0.031 |
| NNC II | 1919 | 1767 | 92.1% |  |
| NNC I ~ NNC II |  |  |  |  |
| NNC I | 1919 | 1726 | 89.9% | 0.021 |
| NNC II | 1919 | 1767 | 92.1% |  |
| ANN I ~ ANN II |  |  |  |  |
| ANN I | 1919 | 1664 | 86.7% | 0.001 |
| ANN II | 1919 | 1729 | 90.1% |  |

ANN: artificial neural network; NNC: neural network cascade; successfully predicted (n): the total number of P450 inhibitors successfully predicted.
