# Supplementary material for: Artificial neural network cascade identifies multi-P450 inhibitors in natural compounds
Source: PeerJ. 2015 Dec 21;3:e1524. doi: 10.7717/peerj.1524 (PMC4696407; doi:10.7717/peerj.1524)
Supplement: Table S9 [file peerj-03-1524-s013.docx]

**Table S9.** Comparison of ANN model II and NNC model II in identifying P450 inhibitors and multi-P450 inhibitors (n = 8148).

| model | compounds (n) | successfully predicted (n) | accuracy | *p* (Chi-squared test) |
| --- | --- | --- | --- | --- |
| P450 inhibitor |  |  |  |  |
| ANN II | 8148 | 6522 | 80.0% | 0.039 |
| NNC II | 8148 | 6626 | 81.3% |  |
| multi-P450 inhibitor |  |  |  |  |
| ANN II | 8148 | 6276 | 77.0% | 0.01 |
| NNC II | 8148 | 6412 | 78.7% |  |

ANN: artificial neural network; NNC: neural network cascade; successfully predicted (n): the total number of compounds that were successfully predicted in term of P450 inhibition.
