# Supplementary material for: Artificial neural network cascade identifies multi-P450 inhibitors in natural compounds
Source: PeerJ. 2015 Dec 21;3:e1524. doi: 10.7717/peerj.1524 (PMC4696407; doi:10.7717/peerj.1524)
Supplement: Table S10 [file peerj-03-1524-s014.docx]

**Table S10.** Prediction result for literature-reported MBIs using NNC model II.

| Chemical | PubChem ID | 1A2 | 2C9 | 2C19 | 2D6 | 3A4 | Reference | PIS | Predicted |
| --- | --- | --- | --- | --- | --- | --- | --- | --- | --- |
| (-)-Clusin | 44575398 | - | - | - | - | MBI | 16248836 | 0.478 | Inhibitor |
| (-)-Dihydroclusin | 332806 | - | - | - | - | MBI | 16248836 | 0.450 | Inhibitor |
| (-)-Dihydrocubebin | 193042 | - | - | - | - | MBI | 16248836 | 0.391 | Inhibitor |
| (-)-Hinokinin | 442879 | - | - | - | - | MBI | 16248836 | 0.504 | Inhibitor |
| (-)-Yatein | 442835 | - | - | - | - | MBI | 16248836 | 0.533 | Inhibitor |
| 17-alpha-Ethynyl estradiol | 5991 | - | - | - | - | MBI | 16248836 | 0.559 | Inhibitor |
| 1-aminobenzotriazole | 1367 | - | - | - | - | MBI | 21722087 | 0.194 | Inhibitor |
| 1-propynyl pyrene | 57396178 | MBI | - | - | - | - | 16248836 | 0.889 | Inhibitor |
| 2-ethynyl naphtalene | 115017 | MBI | - | - | - | - | 16248836 | 0.371 | Inhibitor |
| 2-propynyl phenanthrene | 10059170 | MBI | - | - | - | - | 16248836 | 0.700 | Inhibitor |
| 3-(3'',4''-dimethoxybenzyl)-2-(3',4'-methylenedioxybenzyl)butyrolactone | - | - | - | - | - | MBI | 18563665 | 0.543 | Inhibitor |
| 3,5-Diethoxycarbonyl-1,4-dihydro-2,6-dimethyl-4-ethylpyridine(DDEP) | 70857 | MBI | MBI | - | - | MBI | 16248836 | 0.156 | Non-inhibitor |
| 3-[2-(2,4,6-trimethylphenyl)thioethyl]-4-methylsydnone(TTMS) | 124145 | MBI | - | - | - | MBI | 16248836 | 0.522 | Inhibitor |
| 4-(1-propynyl)biphenyl | 12136078 | MBI | - | - | - | - | 16248836 | 0.767 | Inhibitor |
| 4-Hydroxypropranolol | 91565 | - | - | - | MBI | - | 17584015 | 0.335 | Inhibitor |
| 4-Ipomeanol | 36284 | - | - | - | - | MBI | 16248836 | 0.125 | Non-inhibitor |
| 4-propynyl biphenyl | 23267244 | MBI | - | - | - | - | 16248836 | 0.797 | Inhibitor |
| 5'-hydroxy-5-hydroxymethyl-4'',5''-methylenedioxy-1,2,3,4-dibenzo-1,3,5-cycloheptatriene | - | - | - | - | - | MBI | 18985859 | 0.593 | Inhibitor |
| 5-hydroxygraveroline | - | - | - | - | MBI | - | 22465502 | 0.499 | Inhibitor |
| 5-Hydroxythiabendazole | 108227 | MBI | - | - | - | - | 19754423 | 0.410 | Inhibitor |
| 5-methoxypsoralen | 2355 | - | - | - | - | MBI | 17988092 | 0.482 | Inhibitor |
| 5-Methylchrysene | 19427 | MBI | - | - | - | - | 19754423 | 0.668 | Inhibitor |
| 6',7'-DihydroxyBergamottin | 12082365 | - | - | - | - | MBI | 16248836 | 0.535 | Inhibitor |
| 7-(4-trifluoromethyl)coumarin propargyl ether | - | - | - | - | - | MBI | 18653744 | 0.572 | Inhibitor |
| 7-coumarin propargyl ether | - | - | - | - | - | MBI | 18653744 | 0.492 | Inhibitor |
| 7-Ethynyl-3,4,8-trimethylcoumarin | - | MBI | - | - | - | - | 22443586 | 0.601 | Inhibitor |
| 7-Ethynyl-3-phenylcoumarin | - | MBI | - | - | - | - | 22443586 | 0.687 | Inhibitor |
| 7-Ethynyl-4-(trifluoromethyl)coumarin | - | MBI | - | - | - | - | 22443586 | 0.562 | Inhibitor |
| 7-Ethynyl-4-methyl-3-phenylcoumarin | - | MBI | - | - | - | - | 22443586 | 0.746 | Inhibitor |
| 7-Ethynyl-4-methylcoumarin | - | MBI | - | - | - | - | 22443586 | 0.532 | Inhibitor |
| 7-Ethynyl-6-methoxycoumarin | - | MBI | - | - | - | - | 22443586 | 0.564 | Inhibitor |
| 7-Ethynylcoumarin | - | MBI | - | - | - | - | 22443586 | 0.528 | Inhibitor |
| a reactive intermediate of dihydralazine | - | MBI | - | - | - | - | 17584015 | 0.620 | Inhibitor |
| Acetylene | 6326 | MBI | - | - | - | - | 16248836 | 0.078 | Non-inhibitor |
| alpha-Naphtoflavone | 11790 | - | - | - | - | MBI | 16248836 | 0.725 | Inhibitor |
| Amprenavir | 65016 | - | - | - | - | MBI | 16248836 | 0.338 | Inhibitor |
| Antofloxacin | 24806573 | MBI | - | - | - | - | 19754423 | 0.093 | Non-inhibitor |
| Azamulin | 3086060 | - | - | - | - | MBI | 16248836 | 0.147 | Non-inhibitor |
| B[a]P | 2336 | MBI | - | - | - | - | 19754423 | 0.526 | Inhibitor |
| benz[a]anthracene | 5954 | MBI | - | - | - | - | 19754423 | 0.568 | Inhibitor |
| benzo[b]fluoranthene | 9153 | MBI | - | - | - | - | 19754423 | 0.534 | Inhibitor |
| benzo[j]fluoranthene | 9152 | MBI | - | - | - | - | 19754423 | 0.526 | Inhibitor |
| Bergamottin | 5471349 | - | - | - | - | MBI | 16248836 | 0.643 | Inhibitor |
| Carbamazepine | 2554 | MBI | - | - | - | - | 19754423 | 0.589 | Inhibitor |
| chalepensin | 128834 | - | - | - | - | MBI | 23257392 | 0.662 | Inhibitor |
| Chrysene | 9171 | MBI | - | - | - | - | 19754423 | 0.571 | Inhibitor |
| Cimetidine | 2756 | - | MBI | MBI | MBI | - | 16248836 | 0.117 | Non-inhibitor |
| Clarithromycin | 84029 | - | - | - | - | MBI | 16248836 | 0.227 | Inhibitor |
| Clopidogrel | 60606 | - | - | MBI | - | - | 16248836 | 0.631 | Inhibitor |
| Clorgyline | 4380 | MBI | - | - | - | MBI | 19754423, 24016115 | 0.452 | Inhibitor |
| Compound 15 | - | - | - | - | MBI | - | 16808005 | 0.394 | Inhibitor |
| Compound 17 | - | - | - | - | MBI | - | 16808005 | 0.420 | Inhibitor |
| Cyclohexylline | 54584617 | MBI | - | - | - | - | 16248836 | 0.051 | Non-inhibitor |
| D617 (Verapamil metabolite) | 53886 | - | - | - | - | MBI | 16248836 | 0.441 | Inhibitor |
| Delavirdine | 5625 | - | - | - | - | MBI | 16248836 | 0.543 | Inhibitor |
| Desethylamiodarone | 104774 | MBI | - | - | - | - | 19754423 | 0.660 | Inhibitor |
| Desmethyl tamoxifen | 3036172 | - | - | - | - | MBI | 16248836 | 0.730 | Inhibitor |
| dibenz[a,j]acridine | 9177 | MBI | - | - | - | - | 19754423 | 0.484 | Inhibitor |
| Diclofenac | 3033 | - | - | - | - | MBI | 16248836 | 0.538 | Inhibitor |
| Dihydralazine | 10230 | MBI | - | - | - | MBI | 16248836 | 0.098 | Non-inhibitor |
| Diltiazem | 39186 | - | - | - | - | MBI | 16248836 | 0.324 | Inhibitor |
| DMBA | 6001 | MBI | - | - | - | - | 19754423 | 0.720 | Inhibitor |
| DMP777 | 177992 | - | - | - | - | MBI | 16248836 | 0.383 | Inhibitor |
| DPC-681 | 3083536 | - | - | - | - | MBI | 16248836 | 0.468 | Inhibitor |
| EMTPP | 2813098 | - | - | - | MBI | MBI | 25273356 | 0.554 | Inhibitor |
| Enoxacin | 3229 | MBI | - | - | - | - | 17584015 | 0.065 | Non-inhibitor |
| Erythromycin | 12560 | - | - | - | - | MBI | 16248836 | 0.203 | Inhibitor |
| Fluoxetine | 3386 | - | - | MBI | - | MBI | 24016115 | 0.467 | Inhibitor |
| Furafylline | 3433 | MBI | - | - | - | - | 16248836 | 0.219 | Inhibitor |
| Furanocoumarin dimer (GF-I-1) | - | - | - | - | - | MBI | 16248836 | 0.332 | Inhibitor |
| Furanocoumarin dimer (GF-I-4) | - | - | - | - | - | MBI | 16248836 | 0.323 | Inhibitor |
| Gestodene | 3033968 | - | - | - | - | MBI | 16248836 | 0.491 | Inhibitor |
| Glabridin | 124052 | - | - | - | - | MBI | 16248836 | 0.621 | Inhibitor |
| Gomisin C | 151529 | - | - | - | - | MBI | 16248836 | 0.528 | Inhibitor |
| helioxanthin | 177023 | - | - | - | - | MBI | 18563665 | 0.596 | Inhibitor |
| Hydrastine | 197835 | - | MBI | - | MBI | MBI | 16248836 | 0.505 | Inhibitor |
| hypophyllanthin | 358902 | - | - | - | - | MBI | 21178301 | 0.485 | Inhibitor |
| Imatinib | 5291 | - | - | - | - | MBI | 22014153 | 0.334 | Inhibitor |
| Indinavir | 5362440 | - | - | - | - | MBI | 16248836 | 0.378 | Inhibitor |
| Irinotecan | 60838 | - | - | - | - | MBI | 16248836 | 0.297 | Inhibitor |
| Isoniazid | 3767 | MBI | - | MBI | - | MBI | 16248836 | 0.040 | Non-inhibitor |
| K11002 | - | - | - | - | - | MBI | 16248836 | 0.601 | Inhibitor |
| L-754,394 | 5481990 | - | - | - | MBI | MBI | 16248836 | 0.521 | Inhibitor |
| lapatinib | 208908 | - | - | - | - | MBI | 20624855 | 0.868 | Inhibitor |
| Laromustine | 3081349 | MBI | - | - | - | - | 19754423 | 0.012 | Non-inhibitor |
| Lilopristone | 13490845 | - | - | - | - | MBI | 16248836 | 0.307 | Inhibitor |
| Limonin | 235284 | - | - | - | - | MBI | 16248836 | 0.142 | Non-inhibitor |
| Lopinavir | 92727 | - | - | - | - | MBI | 16248836 | 0.522 | Inhibitor |
| lunamarine | 442922 | - | - | - | MBI | - | 22465502 | 0.575 | Inhibitor |
| madecassoside | 45356919 | - | - | MBI | - | MBI | 21349323 | 0.130 | Non-inhibitor |
| Methimazole | 1349907 | - | MBI | MBI | - | MBI | 16248836 | 0.029 | Non-inhibitor |
| Methylenedioxy methamphetamine | 1615 | - | - | - | MBI | - | 16248836 | 0.251 | Inhibitor |
| Methylenedioxyphenylbenzothiazoline | 54587576 | - | - | - | - | MBI | 16248836 | 0.578 | Inhibitor |
| Metoclopramide | 4168 | - | - | - | MBI | - | 16248836 | 0.170 | Non-inhibitor |
| Mibefradil | 60663 | - | - | - | - | MBI | 16248836 | 0.537 | Inhibitor |
| midazolam | 4192 | - | - | - | - | MBI | 23282066 | 0.802 | Inhibitor |
| Mifepristone | 55245 | - | - | - | - | MBI | 16248836 | 0.467 | Inhibitor |
| MS-PPOH | 35025892 | - | MBI | - | - | - | 21460231 | 0.436 | Inhibitor |
| N-desmethyldiltiazem | 107891 | - | - | - | - | MBI | 16248836 | 0.334 | Inhibitor |
| N-Desmethyltamoxifen | 3032890 | - | - | - | - | MBI | 18473851 | 0.720 | Inhibitor |
| Nefazodone | 4449 | - | - | - | - | MBI | 16248836 | 0.333 | Inhibitor |
| Nelfinavir | 64143 | - | - | - | - | MBI | 16248836 | 0.415 | Inhibitor |
| Nicardipine | 4474 | - | - | - | - | MBI | 18473851 | 0.497 | Inhibitor |
| NNC55-0396 | 9957280 | - | - | - | - | MBI | 18411403 | 0.532 | Inhibitor |
| Oleuropein | 5281544 | - | - | - | - | MBI | 18473851 | 0.093 | Non-inhibitor |
| Oltipraz | 47318 | MBI | - | - | - | - | 16248836 | 0.429 | Inhibitor |
| Onapristone | 5311505 | - | - | - | - | MBI | 16248836 | 0.313 | Inhibitor |
| OSI-930 | 9868037 | - | - | - | - | MBI | 21068193 | 0.720 | Inhibitor |
| Parathion | 991 | - | MBI | - | - | MBI | 16248836 | 0.375 | Inhibitor |
| Paroxetine | 43815 | - | - | - | MBI | MBI | 17584015 | 0.436 | Inhibitor |
| phyllanthin | 358901 | - | - | - | - | MBI | 21178301 | 0.527 | Inhibitor |
| PPOH | 53741002 | - | MBI | - | - | - | 21460231 | 0.439 | Inhibitor |
| R,S-Norverapamil | 104972 | - | - | - | - | MBI | 16248836 | 0.537 | Inhibitor |
| Raloxifene | 5035 | - | - | - | - | MBI | 16248836 | 0.680 | Inhibitor |
| resveratrol | 445154 | MBI | - | - | - | MBI | 17584015 | 0.520 | Inhibitor |
| Retrorsine | 5281743 | - | - | - | - | MBI | 19818743 | 0.021 | Non-inhibitor |
| Ritonavir | 392622 | - | - | - | - | MBI | 16248836 | 0.571 | Inhibitor |
| RO115-1954 | - | - | - | - | MBI | - | 16248836 | 0.330 | Inhibitor |
| Rofecoxib | 5090 | MBI | - | - | - | - | 17584015 | 0.539 | Inhibitor |
| Rutaecarpine | 65752 | - | - | - | - | MBI | 16248836 | 0.764 | Inhibitor |
| Saquinavir | 441243 | - | - | - | - | MBI | 16248836 | 0.262 | Inhibitor |
| savinin | 5281867 | - | - | - | - | MBI | 18563665 | 0.572 | Inhibitor |
| SCH-66712 | 54587574 | - | - | - | MBI | MBI | 25273356 | 0.413 | Inhibitor |
| sertraline | 68617 | - | - | - | - | MBI | 23929629 | 0.799 | Inhibitor |
| sesamin | 72307 | - | MBI | - | - | - | 20851877 | 0.443 | Inhibitor |
| Silybin | 5213 | - | MBI | - | - | MBI | 17584015 | 0.293 | Inhibitor |
| SN-38 | 104842 | - | - | - | - | MBI | 16248836 | 0.313 | Inhibitor |
| Suprofen | 5359 | - | MBI | - | - | - | 16248836 | 0.210 | Inhibitor |
| Sydnone | 198878 | MBI | - | - | - | MBI | 16248836 | 0.294 | Inhibitor |
| Tabimorelin | 9810101 | - | - | - | - | MBI | 18473851 | 0.504 | Inhibitor |
| Tacrine | 1935 | MBI | - | - | - | - | 16248836 | 0.520 | Inhibitor |
| Tadalafil | 110635 | - | - | - | MBI | MBI | 17584015 | 0.404 | Inhibitor |
| Tamoxifen | 2733526 | - | - | - | - | MBI | 16248836 | 0.702 | Inhibitor |
| Thiabendazole | 5430 | MBI | - | - | - | - | 19754423 | 0.432 | Inhibitor |
| ThioTEPA | 5453 | - | - | - | - | MBI | 18473851 | 0.081 | Non-inhibitor |
| thujopsene | 97829 | - | MBI | MBI | - | - | 25343299 | 0.144 | Non-inhibitor |
| Tiamulin | 656958 | - | - | - | - | MBI | 16248836 | 0.243 | Inhibitor |
| Ticlopidine | 5472 | - | - | MBI | - | - | 16248836 | 0.706 | Inhibitor |
| Tienilic acid | 38409 | - | MBI | - | - | - | 16248836 | 0.343 | Inhibitor |
| troglitazone | 5591 | - | - | - | - | MBI | 17584015 | 0.686 | Inhibitor |
| Troleandomycin | 202225 | - | - | - | - | MBI | 16248836 | 0.216 | Inhibitor |
| Verapamil | 2520 | - | - | - | - | MBI | 16248836 | 0.516 | Inhibitor |
| Zafirlukast | 5717 | - | - | - | - | MBI | 17584015 | 0.725 | Inhibitor |
| Zileuton | 10681296 | MBI | - | - | - | - | 16248836 | 0.298 | Inhibitor |
| Zolpidem | 5732 | - | - | - | - | MBI | 17598095 | 0.733 | Inhibitor |

-: not available; MBI: mechanism-based inhibitor; PIS: P450 inhibition score.
